# In-Situ H_2_O_2_ Cleaning for Fouling Control of Manganese-Doped Ceramic Membrane through Confined Catalytic Oxidation Inside Membrane

**DOI:** 10.3390/membranes12010021

**Published:** 2021-12-24

**Authors:** Shengyin Tang, Wanyi Fu, Tiantian Song, Tianhao Tang, Li Chen, Jianning Guo, Slav W. Hermanowicz, Xihui Zhang

**Affiliations:** 1 Tsinghua-Berkeley Shenzhen Institute, Tsinghua University, Shenzhen 518055, China; sherrt3@uci.edu (S.T.); 15733948667@163.com (T.S.); TianhaoTang_THU@163.com (T.T.); 2 Tsinghua-Berkeley Shenzhen Institute, University of California, Berkeley, CA 94720, USA; hermanowicz@ceeucb.com; 3 Tsinghua Shenzhen International Graduate School, Tsinghua University, Shenzhen 518055, China; fenglin1129@163.com; 4 Institute of Information Technology, Shenzhen 518172, China; guojn08@163.com

**Keywords:** manganese-doped ceramic membrane, fouling mitigation, in-situ cleaning, hydrogen peroxide, confined catalytic oxidation, wastewater treatment

## Abstract

This work presents an effective approach for manganese-doped Al_2_O_3_ ceramic membrane (Mn-doped membrane) fouling control by in-situ confined H_2_O_2_ cleaning in wastewater treatment. An Mn-doped membrane with 0.7 atomic percent Mn doping in the membrane layer was used in a membrane bioreactor with the aim to improve the catalytic activity toward oxidation of foulants by H_2_O_2_. Backwashing with 1 mM H_2_O_2_ solution at a flux of 120 L/m^2^/h (LMH) for 1 min was determined to be the optimal mode for in-situ H_2_O_2_ cleaning, with confined H_2_O_2_ decomposition inside the membrane. The Mn-doped membrane with in-situ H_2_O_2_ cleaning demonstrated much better fouling mitigation efficiency than a pristine Al_2_O_3_ ceramic membrane (pristine membrane). With in-situ H_2_O_2_ cleaning, the transmembrane pressure increase (ΔTMP) of the Mn-doped membrane was 22.2 kPa after 24-h filtration, which was 40.5% lower than that of the pristine membrane (37.3 kPa). The enhanced fouling mitigation was attributed to Mn doping, in the Mn-doped membrane layer, that improved the membrane surface properties and confined the catalytic oxidation of foulants by H_2_O_2_ inside the membrane. Mn^3+^/Mn^4+^ redox couples in the Mn-doped membrane catalyzed H_2_O_2_ decomposition continuously to generate reactive oxygen species (ROS) (i.e., HO• and O_2_^1^), which were likely to be confined in membrane pores and efficiently degraded organic foulants.

## 1. Introduction

Ceramic membranes have been widely used in the membrane bioreactor (MBR) for wastewater treatment [1]. However, membrane fouling remains a challenge for the wide application of ceramic membrane bioreactors [2]. During membrane filtration, organic foulants in wastewater gradually accumulate on the ceramic membrane surface and block membrane pores, causing severe membrane fouling [3]. To control membrane fouling, membrane cleaning is an essential part of the maintenance of MBR (e.g., backwashing, forward washing and ex-situ cleaning). Fouled membranes are commonly immersed in sodium hypochlorite (NaClO) solutions to remove foulants from membranes in municipal wastewater treatment [4]. However, ex-situ cleaning requires long suspension of MBR, large consumption of chemicals and complex operations. With the aim to reduce the frequency of ex-situ cleaning, in-situ chemical cleaning via backwashing with chemical solutions is employed during filtration [5]. However, traditional in-situ backwashing with chemical agents (e.g., NaClO) poses a high risk of harming the microorganisms in active sludge when the oxidants are backwashed into a mixed liquor [6]. Thus, it is interesting to explore an approach to effectively control membrane fouling but with no harm to microbes and requiring only a short stop time.

On the other hand, membrane surface modification has been studied as a means to mitigate membrane fouling [7,8,9]. With the advantages of strong catalytic activity and a low price, manganese oxides have been considered promising additives for membrane modification [10,11,12]. Byun et al. reported that a ZrO_2_ ceramic membrane coated with manganese oxide on its surfaces demonstrated a higher anti-fouling tendency during the filtration of real surface water than membranes coated with TiO_2_ or Fe_2_O_3_ [13]. Moreover, manganese oxides have been studied as catalysts and activators for oxidants, such as ozone (O_3_), peroxymonosulfate (PMS) and hydrogen peroxide (H_2_O_2_), in advanced oxidation processes to generate reactive oxygen species (e.g., hydroxyl radical (HO•), superoxide radical (O_2_•^−^), and sulfate radical (SO_4_•^−^)), which further promote the removal of organic foulants [14,15,16]. Compared with O_3_ and PMS, H_2_O_2_ has a higher water solubility than O_3_ and constitutes a green and environmentally friendly oxidant because it releases only water as its by-product [17]. Thus, manganese oxide-doped Al_2_O_3_ ceramic membrane coupling with in-situ H_2_O_2_ cleaning is expected to enhance the removal of organic foulants, providing a novel strategy to control membrane fouling. 

Another issue regarding in-situ chemical cleaning is the potential harm from the oxidants to the functional bacteria in activated sludge [18]. In order to avoid the undesirable harm to activated sludge, H_2_O_2_ solution could be pumped into the membrane through the inner cavity to control the catalytic oxidation of organic foulants occurring within membrane pores. Confined spaces in membrane pores could achieve catalytic performances that are orders of magnitude faster than those obtained in the bulk phase [19]. We found that the ozone decomposition rate inside the membrane pores was about 428 times faster than that in the bulk phase, which was confirmed as a confinement effect of nano-scale membrane pores [20]. Similarly, it is believed that the confinement effect toward catalytic decomposition of H_2_O_2_ within membrane pores could also promote the oxidation of foulants and enhance the cleaning efficiency. Overall, doping manganese oxide in an Al_2_O_3_ ceramic membrane active layer is expected to bring about a double-win effect, both enhancing the membrane antifouling performance and promoting fouling mitigation via catalytic oxidation of organic foulants by H_2_O_2_ within membrane pores.

In this study, an Al_2_O_3_ ceramic membrane doped with Mn_2_O_3_ in the membrane active layer (Mn-doped membrane) was applied in MBR for the treatment of real municipal wastewater. The fouling mitigation behavior of the Mn-doped membrane during in-situ H_2_O_2_ cleaning confined in membrane pores through the inner cavity was investigated and compared with the conventional Al_2_O_3_ ceramic membrane (pristine membrane). Furthermore, we discuss the fouling mitigation mechanisms in terms of surface properties, catalytic activity and the confinement effect of Mn-doped catalytic membranes.

## 2. Materials and Methods

### 2.1. Ceramic Membranes and Characterizations

The manganese-doped Al_2_O_3_ ceramic membrane (Mn-doped membrane) and Al_2_O_3_ ceramic membrane (pristine membrane) were provided by Shenzhen Huayuan Environmental Technology Co., Ltd, Shenzhen, China. Both membranes were composed of a 0.36 mm-thick active layer and 1.1 mm-thick supporting layer. The substrate (supporting layer) of both the Mn-doped membrane and the pristine membrane was made of α-Al_2_O_3_ and SiO_2_. The membrane active layer of the Mn-doped membrane was made of 2% Mn_2_O_3_ and 98% α-Al_2_O_3_. The pristine membrane was made by the same procedure, without the addition of any manganese oxides.

The surface morphologies and elemental compositions of ceramic membranes were analyzed by a scanning electron microscope combined with an energy dispersive spectrometer (SEM-EDS, SU8010, Hitachi, Tokyo, Japan). The XPS spectra of membrane surfaces were detected by X-ray photoelectron spectroscopy (XPS, PHI 5000 Versa Probe II, ULVAC-PHI, Chigasaki, Japan) using an Al Kα X-ray source. 

Catalytic activities of the pristine and Mn-doped membranes toward H_2_O_2_ were characterized by the decomposition efficiency of H_2_O_2_. To do so, 1 L of 1 mM H_2_O_2_ solution was circularly filtrated with ceramic membranes under a membrane flux of 60 LMH. The catalytic stability of membranes was analyzed by cycle tests. For each cycle test, membranes were cleaned by being immersed in 1 L NaClO solution (1000 ppm available chlorine) for 24 h, then immersed in 1 L pure water for another 24 h and finally dried in an oven at 105 °C before the tests. All cycle tests were run in duplicate under a room temperature ranging from 25–28 °C. 

The H_2_O_2_ concentration was analyzed by the spectrophotometric-iodide method [21]. Free radicals generated in 1 mM H_2_O_2_ solution catalyzed by the pristine and Mn-doped membranes were detected by electron paramagnetic resonance (EPR, BRUKER ER070, Karlsruhe, Germany) using 5,5-dimethyl-1-pyrroline N-oxide (DMPO) as the spin trapping agent [22].

### 2.2. Membrane Bioreactor Filtration Tests

#### 2.2.1. Experimental Setup

**Figure 1:**
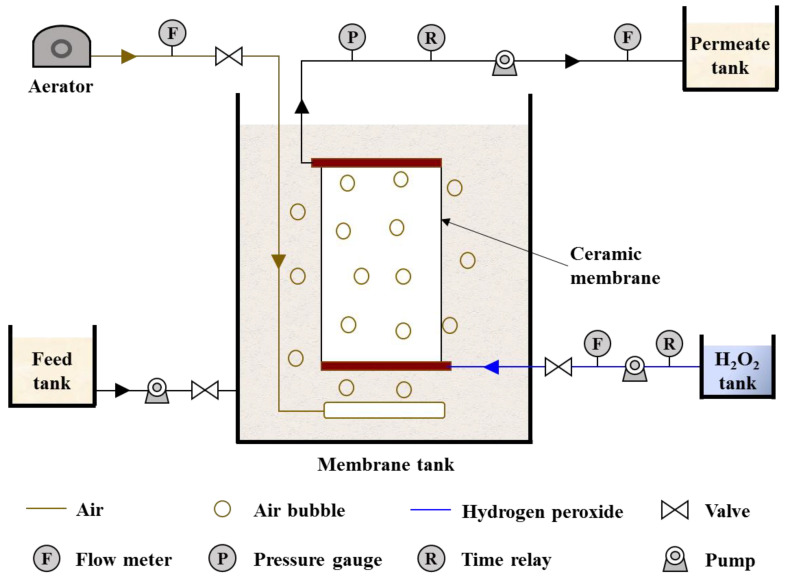
Schematic diagram of experimental setup.

As shown in Figure 1, the MBR system consisted of membrane modules, an air aerator and pumps for feed, permeation and cleaning. The membrane tank was 20 cm × 8 cm × 50 cm (L × W × H) and the active volume was 8 L. The surface area of the ceramic membrane was 0.044 m^2^. The ceramic membrane had two outlets in the diagonal. One was connected with the permeation pump and the other was connected with the chemical feeding pump. The latter outlet was closed during membrane filtration and opened during the chemical cleaning processes. During membrane filtration, an aerator provided air aeration for sufficient mixing of liquor in the membrane tank with an aeration rate of 21 L/h.

#### 2.2.2. Optimal In-Situ H_2_O_2_ Cleaning Mode Tests

To prevent H_2_O_2_ from entering the membrane tank and damaging the activated sludge during in-situ H_2_O_2_ cleaning, the optimal dosing mode of H_2_O_2_ was explored. The effect of the in-situ H_2_O_2_ cleaning mode (i.e., backwash flux and H_2_O_2_ dosage) on the concentration of H_2_O_2_ in the membrane tank was studied in pure water tests (see experimental setup in Figure 1). The pristine or Mn-doped membranes were vertically fixed within the membrane tank. Prior to membrane filtration, 6 L of pure water (same volume with the fed mixed liquor during membrane fouling test) was fed into the membrane tank. Membrane filtration with pure water was conducted under a flux of 60 LMH for more than 10 min prior to backwashing. After the membrane inner cavity was fully filled with pure water, H_2_O_2_ backwash tests were conducted under different modes (i.e., backwashing of H_2_O_2_ solution with dosages of 1, 3 or 5 mM at a flux of 120 LMH for 1 min or backwashing of 1 mM H_2_O_2_ solution at a flux of 120 or 180 LMH for 1 min). H_2_O_2_ solution was backwashed into membranes through the inner cavity by a cleaning pump, while the feed and permeation pumps were closed. At 0, 1, 2, 5, 10, 15, 20 and 30 min of backwashing, water samples were collected from areas near both sides of the ceramic membrane surface using the five-spot sampling method, and the concentration of H_2_O_2_ in the water samples was measured. All experiments were conducted in triplicate. In this study, backwashing with 1 mM H_2_O_2_ at a flux of 120 LMH for 1 min was determined to be the optimal dosing mode, which resulted in undetected H_2_O_2_ in the membrane tank during backwashing.

#### 2.2.3. Fouling Mitigation Tests

The feed of the ceramic membrane reactor was the supernatant of fresh mixed liquor sampled from the aerobic tank in a municipal wastewater treatment plant in Shenzhen, China. The mixed liquid suspended solids (MLSS) of the initial mixed liquor fed into the membrane tank was 6.8 ± 0.1 g/L and the zeta potential was −12.9 ± 1.0 mV. The sludge volume after 30 min sedimentation (SV_30_) of active sludge was 38.8 ± 1.5%. 

Membrane filtration was conducted at a constant flux of 60 LMH with an on/off ratio of 9 min/1 min. The on/off filtration mode was accurately controlled by time relays. A pressure gauge measured and recorded the real-time transmembrane pressure (TMP). 

For the membrane fouling mitigation, two kinds of intermittent in-situ cleaning strategies (clean with H_2_O or 1 mM H_2_O_2_) were applied to evaluate the mitigation efficiencies of the pristine membrane and Mn-doped membrane. H_2_O or 1 mM H_2_O_2_ solution was regularly pumped into ceramic membrane pores every six hours under a flux of 120 LMH for 1 min, and then H_2_O or H_2_O_2_ reacted with organic foulants within membrane pores for 10 min after each clean. TMP variations during the membrane filtration were recorded and used as the indicator of membrane fouling. The TMP recovery ratio (*R*) after each clean was calculated using Equation (1). All filtration experiments ran for 24 h in duplicate under a room temperature ranging from 20–25 °C.

(1) $$R\;(\%)=\frac{{\mathrm{TMP}}_{\mathrm{before}\;\mathrm{cleaning}}-{\mathrm{TMP}}_{\mathrm{after}\;\mathrm{cleaning}}}{{\mathrm{TMP}}_{\mathrm{before}\;\mathrm{cleaning}}}\times 100$$

After the filtration experiment, the fouled membranes were rinsed with 100 mL ultrapure water, and then soaked in 500 mL NaClO solution (1000 ppm available chlorine) for 24 h [23,24]. The size-fractionated (≤0.45 μm) cleaning solutions were then analyzed for dissolved organic carbon (DOC). In addition, membrane permeates were collected every six hours during membrane filtration for analysis of water quality (i.e., turbidity, DOC, total nitrogen and total phosphorus). Mixed liquor properties (i.e., MLSS, SV_30_, zeta potential, specific oxygen uptake rate (SOUR) and DOC) were analyzed after membrane filtration. Detailed analytic methods can be found in our recent papers [23,24]. 

## 3. Results and Discussions

### 3.1. Characterization of Ceramic Membranes

**Figure 2:**
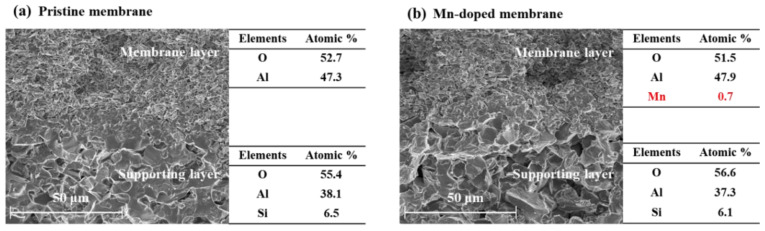
The cross-section morphologies and element compositions of the pristine membrane and Mn-doped membrane.

**Figure 3:**
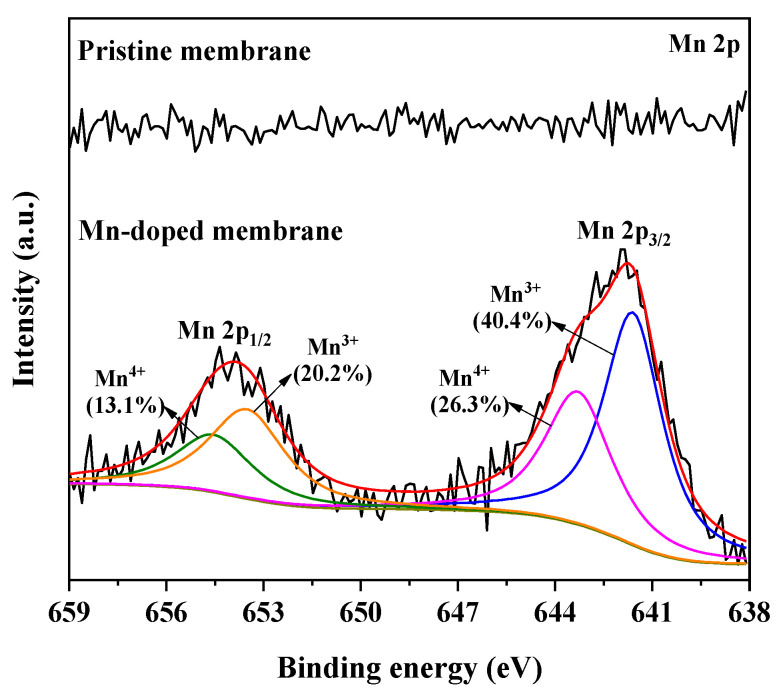
XPS spectra of Mn 2p of the pristine and Mn-doped membranes.

In this study, the Mn element was successfully doped into the membrane layer of the Mn-doped membrane with an atomic percent of 0.7% (Figure 2). The element compositions in supporting layers of the pristine membrane and Mn-doped membrane were almost the same. The average pore sizes of the pristine and Mn-doped membrane active layers were 780 nm (Figure S1a) and 792 nm (Figure S1b), respectively. As shown in Figure 3, the Mn 2p_3/2_ spectrum showed two distinct peaks at 641.6 eV and 643.2 eV in the Mn-doped membrane layer, which were consistent with the binding energy of Mn(III) and Mn(IV) [25,26,27]. This demonstrated that the doped manganese in the Mn-doped membrane existed as two oxidation states (Mn^3+^ and Mn^4+^). The existence of Mn^3+^/Mn^4+^ couples would be beneficial for the catalytic activity of Mn-doped membranes [28].

### 3.2. Catalytic Activity of Ceramic Membranes in H_2_O_2_ Decomposition

**Figure 4:**
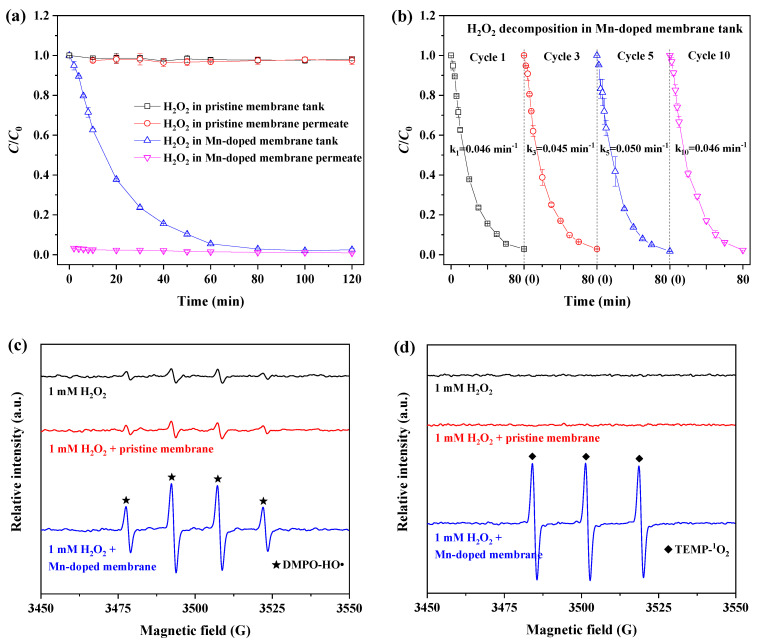
Catalytic activity of (**a**) fabricated ceramic membranes in H_2_O_2_ decomposition and (**b**) an Mn-doped membrane cleaned with 1000 ppm NaClO solution (operating conditions: *C*_0_ = [H_2_O_2_]_0_ = 1 mM; *C* is the concentration of H_2_O_2_ in the membrane tank or permeation; membrane flux = 60 LMH); EPR spectra of (**c**) DMPO-HO• and (**d**) TEMP-^1^O_2_ in 1 mM H_2_O_2_ solution catalyzed by a pristine or Mn-doped membrane.

Figure 4 shows the variation in H_2_O_2_ concentration with respect to filtration time with the pristine and Mn-doped membranes in a circular mode. As shown in Figure 4a, less than 1% of H_2_O_2_ was decomposed throughout two hours of filtration by the pristine membrane. This indicated that the pristine membrane caused negligible improvement in H_2_O_2_ decomposition because of the non-catalytic activity of Al_2_O_3_ and SiO_2_ powders toward H_2_O_2_ decomposition (Figure S2a). 

In comparison, more than 98% of H_2_O_2_ was constantly decomposed in permeation of the Mn-doped membrane, and the concentration of H_2_O_2_ was lower than 0.03 mM. The concentration of H_2_O_2_ in the tank with the Mn-doped membrane gradually decreased with increasing filtration time, and the decomposition efficiency of H_2_O_2_ solution reached 94.6% after one hour of filtration and 97.5% after two hours of filtration. Obviously, the Mn-doped membrane exhibited excellent catalytic activity toward H_2_O_2_ decomposition. This was consistent with the finding that Mn_2_O_3_ power had very high catalytic activity when it came to H_2_O_2_ decomposition (Figure S2b). Moreover, the catalytic activity of the Mn-doped membrane for H_2_O_2_ decomposition did not deteriorate after chemical cleaning with NaClO (1000 ppm) (Figure 4b). The decomposition of H_2_O_2_ in the tank with the Mn-doped membrane followed first-order kinetics, and the average observed rate constant was 4.6 × 10^−2^ min^−1^. There was no significant difference in the observed rate constants for the first, third, fifth and tenth cycle tests. During the chemical cleaning with NaClO (1000 ppm, pH > 9), the concentration of Mn in the NaClO immersion solution was below the detection limit (0.001 mg/L) of flame atomic absorption spectrometry. Mn leaching was not significantly observed during membrane chemical cleaning with NaClO solution at an alkaline pH. These results indicate the high catalytic activity and stability of the Mn-doped membrane when it came to H_2_O_2_ decomposition.

It has been reported that ROS are generated from H_2_O_2_ decomposition. EPR tests were conducted to confirm the generation of ROS from H_2_O_2_ decomposition catalyzed by the pristine and Mn-doped membranes. Four characteristic peaks of DMPO-HO• with an intensity ratio of 1:2:2:1 (Figure 4c) were observed in 1 mM H_2_O_2_ solution without catalysis of the pristine or Mn-doped membranes as a result of the self-decomposition of H_2_O_2_ at a neutral pH [29,30]. There was no obvious difference in the peak intensities of the DMPO-HO• spectra between the H_2_O_2_ solutions with and without the catalysis of the pristine membrane. This result confirmed that the pristine membrane had no catalytic effect on promoting the generation of ROS, consistent with the results of H_2_O_2_ decomposition (Figure 4a). 

The peak intensities of DMPO-HO• in 1 mM H_2_O_2_ solution catalyzed by the Mn-doped membrane were much higher than those in H_2_O_2_ solutions with and without catalysis of the pristine membrane (Figure 4c). Moreover, three characteristic peaks of TEMP-^1^O_2_ with an intensity ratio of 1:1:1 were significantly observed in a 1 mM H_2_O_2_ solution catalyzed by the Mn-doped membrane [31] (Figure 4d), while these were not detectable in 1 mM H_2_O_2_ solutions with or without catalysis of the pristine membrane. These results demonstrated the generation and coexistence of HO• and ^1^O_2_ in H_2_O_2_ solution catalyzed by the Mn-doped membrane [32,33]. This finding directly suggested that Mn doping is a good strategy for enhancing ceramic membrane catalytic activity toward H_2_O_2_, thus decomposing H_2_O_2_ into more powerful ROS, namely HO• or ^1^O_2_ [34].

### 3.3. Determination of Optimal H_2_O_2_ Cleaning Mode for Filtration

**Figure 5:**
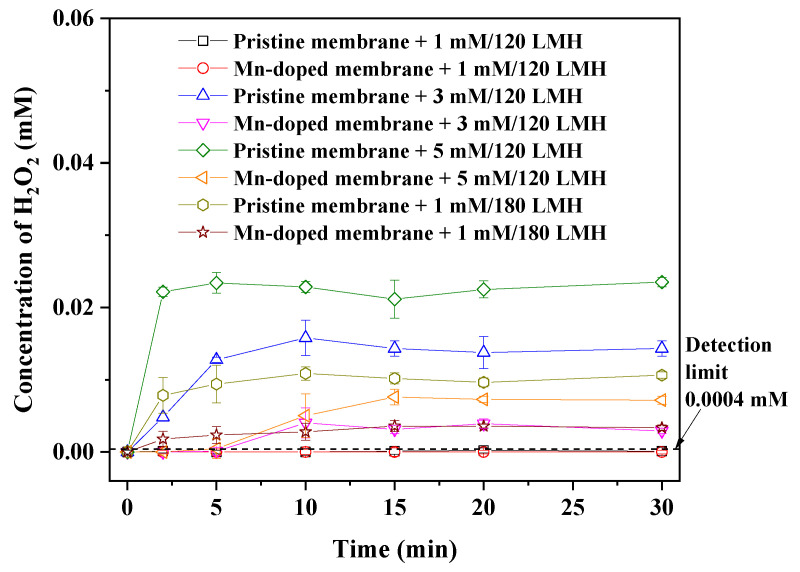
Effect of H_2_O_2_ backwash mode on the concentration of H_2_O_2_ in the membrane tank.

As shown in Figure 5, residual H_2_O_2_ was detected in the membrane tank when the pristine and Mn-doped membranes were backwashed with H_2_O_2_ solution for 1 min under 3 or 5 mM H_2_O_2_/120 LMH or 1 mM H_2_O_2_/180 LMH. This result indicated that residual H_2_O_2_ entered the membrane tank through membrane pores after over-dosage of H_2_O_2_, and thus resulted in an increasing H_2_O_2_ concentration in the membrane tank. The H_2_O_2_ concentration in the membrane tank increased with increasing H_2_O_2_ dosage and backwash flux, resulting in an increased risk of harm to activated sludge. Under the same dosing mode of H_2_O_2_, H_2_O_2_ concentrations in the Mn-doped membrane tank were always much lower than those in the pristine membrane tank. The lower H_2_O_2_ concentration in the Mn-doped membrane tank was attributed to the high catalytic activity of the Mn-doped membrane toward H_2_O_2_ decomposition (Figure 4). This result demonstrated that Mn doping could reduce the risk of damaging the activated sludge during in-situ H_2_O_2_ backwashing by accelerating H_2_O_2_ decomposition within the Mn-doped membrane pores.

When the pristine and Mn-doped membranes were backwashed with 1 mM H_2_O_2_ at a flux of 120 LMH for 1 min, the concentrations of H_2_O_2_ in the pristine and Mn-doped membrane tanks were all below the detection limit (0.0004 mM) of the spectrophotometric-iodide method. Obviously, almost all H_2_O_2_ was confined within the membrane under this dosing mode, causing an undetectable change in the H_2_O_2_ concentration in the membrane tank (volume of 6 L). Therefore, with the aim to protect activated sludge from being damaged by residual H_2_O_2_ in the membrane tank, we conducted backwashing with 1 mM H_2_O_2_ at a flux of 120 LMH for 1 min as the optimal dosing mode for in-situ confined H_2_O_2_ cleaning.

### 3.4. Membrane Fouling Mitigation Performance with and without In-Situ H_2_O_2_ Cleaning

**Figure 6:**
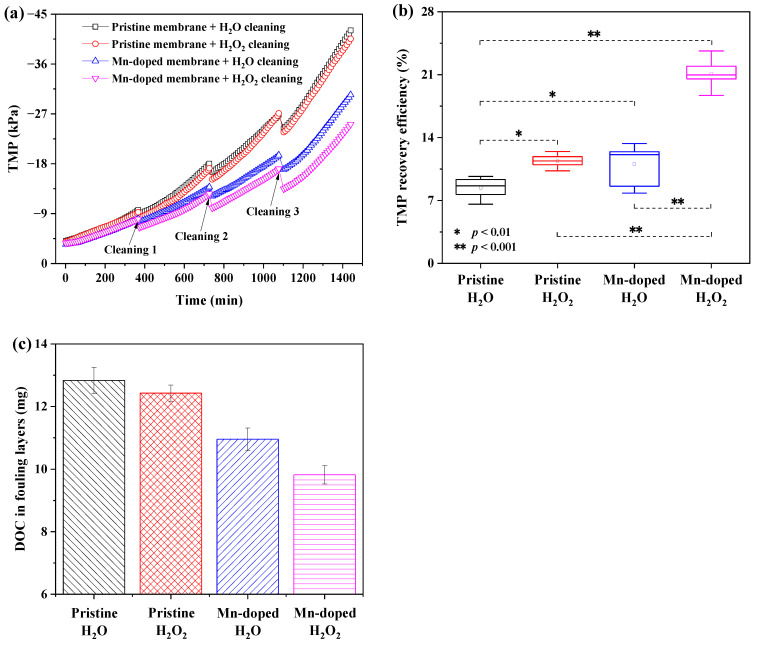
Membrane fouling mitigation behaviors of different membranes coupling with different in-situ cleaning methods. (**a**) TMP during filtration and cleaning; (**b**) TMP recovery efficiencies after cleaning; (**c**) the total content of DOC in membrane fouling layers after 24 h filtration.

As seen from Figure 6a, the Mn-doped membrane demonstrated an obviously lower TMP increase rate than the pristine membrane. The TMP of the pristine membrane with H_2_O cleaning rapidly increased to −42.1 kPa after 24 h of filtration, while the TMP of the Mn-doped membrane with H_2_O cleaning only increased to −30.4 kPa. After membrane filtration, the total DOC content in the fouling layers of the Mn-doped membrane with H_2_O cleaning (11.0 mg) was also lower than that of the pristine membrane with H_2_O cleaning (12.8 mg) (Figure 6c). These results demonstrated the improved antifouling properties of the Mn-doped membrane. With H_2_O_2_ cleaning, the TMP of the pristine membrane increased to −40.5 kPa after 24-h filtration, while the TMP of the Mn-doped membrane increased to −25.1 kPa. The total DOC content in the fouling layers decreased from 12.4 mg (the pristine membrane with H_2_O_2_ cleaning) to 9.8 mg (the Mn-doped membrane with H_2_O_2_ cleaning) (Figure 6c). This suggested that intermittent in-situ H_2_O_2_ cleaning efficiently controlled fouling of the Mn-doped membrane but had a slight positive effect on fouling mitigation of the pristine membrane.

The TMP recovery ratio (*R*) achieved by each cleaning mode was calculated to investigate the efficiencies of different cleaning strategies. As shown in Figure 6b, the Mn-doped membrane exhibited the highest average TMP recovery efficiency (21.1 ± 1.4%) with H_2_O_2_ cleaning, compared to that with H_2_O cleaning (11.1 ± 2.5%), the pristine membrane with H_2_O (8.4 ± 1.2%) or H_2_O_2_ cleaning (11.4 ± 0.8%). The average TMP recovery efficiencies of membranes with H_2_O_2_ cleaning were significantly higher (*p* < 0.01) than those of membranes with H_2_O cleaning, indicating that oxidation by H_2_O_2_ successfully removed foulants from membranes. Moreover, the Mn-doped membrane with H_2_O_2_ cleaning achieved 85.1% higher TMP recovery efficiency than the pristine membrane with H_2_O_2_ cleaning. Obviously, Mn doping in the Mn-doped membrane catalyzed the oxidation of organic foulants by H_2_O_2_ within the membrane. It is interesting to note that the average TMP recovery efficiency of the Mn-doped membrane with H_2_O cleaning (11.1 ± 2.5 %) and that of the pristine membrane with H_2_O_2_ cleaning (11.4 ± 0.8%) had no significant difference (Figure 6b), but the overall TMP increase and the total DOC content in membrane fouling layers of the former (ΔTMP = 27.7 kPa in 24 h, the total content of DOC = 11.0 mg) were much lower than of the latter (ΔTMP = 37.3 kPa, the total content of DOC = 12.4 mg). This further proved that antifouling properties of membranes were more critical than the cleaning strategy for fouling mitigation when it came to membranes without catalytic activity toward H_2_O_2_. 

It is worth noting that the H_2_O_2_ concentrations in both the pristine membrane and Mn-doped membrane tank were below the detection limit (<0.0004 mM) after in-situ H_2_O_2_ cleaning. This result demonstrated that in-situ H_2_O_2_ cleaning with the optimal mode of 1 mM H_2_O_2_ at a flux of 120 LMH for 1 min caused an undetectable change in the H_2_O_2_ concentration in the membrane tank, which was consistent with the results of pure water tests (Figure 5). Moreover, there was no obvious difference of mixed liquor properties (i.e., DOC, MLSS, SV_30_, zeta potential, SOUR, pH) between four membrane bioreactors tested with H_2_O or H_2_O_2_ cleaning (Table S1). This verified that in-situ H_2_O_2_ cleaning with the optimal mode did not affect the mixed liquor properties in the membrane bioreactor. Activated sludge of MBRs was well-protected from being damaged by H_2_O_2_. The water quality (i.e., turbidity, DOC, total nitrogen and total phosphorus) of permeation (Figure S3) also showed no significant difference between the pristine membrane and Mn-doped membrane with H_2_O or H_2_O_2_ cleaning.

Overall, coupling the Mn-doped membrane and intermittent in-situ H_2_O_2_ cleaning achieved the best fouling mitigation performance with the lowest TMP increase and the highest TMP recovery efficiency, causing no harm to the activated sludge.

### 3.5. Fouling Mitigation Mechanisms of Mn-Doped Membrane with In-Situ H2O2 Cleaning

#### 3.5.1. Enhanced Antifouling Properties of Mn-Doped Membrane

All the results indicated that the Mn-doped membrane exhibited a better antifouling performance than the pristine membrane during filtration of real municipal wastewater. This was attributed to the modified surface properties after Mn doping in the membrane layer, including an intensified electric charge and heightened hydrophilicity [35]. In this study, the pristine membrane layer consisted of Al_2_O_3_ that was positively charged at pH 7.0, while the Mn-doped membrane layer was doped with Mn_2_O_3_ that was negatively charged at the same pH. Mn doping changed the membrane surface charge from positive (pristine membrane) to negative (Mn-doped membrane). Organic foulants, especially biopolymers, were the main contributors to membrane fouling as a result of the high rejection of biopolymers by the pristine and Mn-doped membranes (Figures S4 and S5). Accordingly, the interactions between organic foulants and the ceramic membrane surface changed significantly after Mn doping. The negatively charged surface of the Mn-doped membrane changed the electrostatic attraction force between organic foulants and the pristine membrane to an electrostatic repulsion force [36,37,38]. This inhibited the adsorption and accumulation of organic foulants on the Mn-doped membrane surfaces and further in the pores. Moreover, the pure water contact angle of the Mn-doped membrane (24.1°) was 24.5% lower than that of the pristine membrane (31.9°) (Figure S6). This meant better surface hydrophilicity was achieved by Mn doping. This was in agreement with the analysis of the O 1s spectrum (Figure S7), which showed a greater amount of hydroxyl groups in the Mn-doped membrane. The increase of hydrogen-bond interactions between hydroxyl groups and water made the Mn-doped membrane more hydrophilic [39,40]. Higher hydrophilicity of the Mn-doped membrane weakened the hydrophobic interaction between organic compounds and membrane surface [41,42]. The biopolymer rejection rate by the Mn-doped membrane (49.0%) was lower than that by the pristine membrane (53.9%). Subsequently, fewer organic foulants attached to membrane/pore surfaces (Figure 6c) during membrane filtration, leading to a lower TMP increase rate and mitigating the Mn-doped membrane fouling [13,43]. Usually, hydrophilic membranes require a lower pressure than hydrophobic membranes to obtain the same permeation flux [44,45,46,47]. This is consistent with the fact that the operation TMP of the Mn-doped membrane was lower than that of the pristine membrane at the same flux (Figure S8). Therefore, with a lower operation TMP and slower TMP increase rate, the final TMP of the Mn-doped membrane with H_2_O cleaning (−30.4 kPa) was 27.8% lower than that of the pristine membrane with H_2_O cleaning (−42.1 kPa) after 24 h of filtration of real municipal wastewater.

#### 3.5.2. Intensified Catalytic Activity of Mn-Doped Membrane toward H_2_O_2_ Decomposition

**Figure 7:**
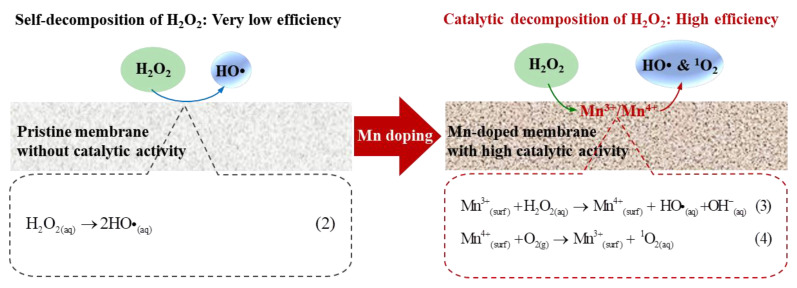
Schematic diagram of H_2_O_2_ decomposition catalyzed by the pristine membrane and Mn-doped membrane.

Coupling the Mn-doped membrane with in-situ H_2_O_2_ cleaning exhibited the highest TMP recovery after H_2_O_2_ cleaning. This was attributed to the enhanced catalytic activity toward H_2_O_2_ decomposition inside the Mn-doped membrane. The existence of Mn^3+^/Mn^4+^ couples (i.e., reversible transition between Mn^3+^ and Mn^4+^) in the Mn-doped membrane layer (Figure 3) played a crucial role in improving the catalytic activity of the membranes toward H_2_O_2_ decomposition and generation of ROS (i.e., HO• and ^1^O_2_) [15,28,48]. When H_2_O_2_ solution was pumped into the Mn-doped membrane, available Mn^3+^/Mn^4+^ couples at the manganese-based catalyst surface effectively catalyzed the decomposition of H_2_O_2_ (>98% of 1 mM H_2_O_2_ decomposed inside membrane pores (Figure 4a,b)) to continuously produce ROS (i.e., HO• and ^1^O_2_ (Figure 4c,d)) (see Equations (3) and (4) in Figure 7) [14,28,33,48]. The oxidizing powers of generated ROS, especially HO•, toward organic matters are much stronger than that of H_2_O_2_ [49,50]. The adsorbed organic foulants can be efficiently oxidized by generated ROS into smaller molecules and finally removed from the Mn-doped membrane. Therefore, the total DOC content in the fouling layers of the Mn-doped membrane decreased from 11.0 mg (with H_2_O cleaning) to 9.8 mg (with H_2_O_2_ cleaning) (Figure 6c). However, the pristine membrane consisted of Al_2_O_3_ and SiO_2_ that had no catalytic activity toward H_2_O_2_ decomposition (Figure S2a). The decomposition efficiency of H_2_O_2_ with the pristine membrane was very low (<1% within two hours) (Figure 4a) and led to a very limited amount of HO• (Figure 4c) mostly from the self-decomposition of 1 mM H_2_O_2_ (see Equation (2) in Figure 7). Consequently, the low decomposition rate of H_2_O_2_ inside the pristine membrane largely limited the oxidation removal efficiency of organic foulants of the fouled pristine membrane during in-situ H_2_O_2_ cleaning with a short stop time (10 min). The total DOC content in the fouling layers of the pristine membrane only decreased from 12.8 mg (with H_2_O cleaning) to 12.4 mg (with H_2_O_2_ cleaning) (Figure 6c). Therefore, the TMP recovery efficiency of the Mn-doped membrane after H_2_O_2_ cleaning was significantly higher than that of the pristine membrane.

#### 3.5.3. Confined Catalytic Oxidation Performance within Mn-Doped Membrane Pores

In addition, H_2_O_2_ solutions were pumped into the membrane and confined inside the membrane pores almost without detectable residual H_2_O_2_ entering the membrane tank. Limited reaction space within the Mn-doped membrane pores further enhanced the oxidation of organic foulants by enriched ROS [51]. It is well-known that the lifetimes of HO• (<1 μs) and ^1^O_2_ (~3 μs) in water are extremely short, resulting in elimination before they react with pollutants [52]. In heterogeneous catalytic reaction systems, the concentration of ROS is the highest on the catalyst surface and then rapidly decreases with increasing diffusion length [19]. Assuming the lifetimes (τ) of HO• and ^1^O_2_ are 1 μs and 3 μs, respectively, at a neutral pH [53], the diffusion lengths (*λ*_*L*_) of HO• and ^1^O_2_ in the aqueous phase should be ~96 nm and ~159 nm, respectively, as calculated by $\lambda_{L}=2\sqrt{\tau \times D}$ (where *D* is the diffusion coefficient; $D_{\mathrm{HO}•}=2.3\times 10^{-9}\;m^{2}\;s^{-1}$ and $D_{{}^{1}O_{2}}=2.1\times 10^{-9}\;m^{2}\;s^{-1}$) [54]. Thus, the oxidation efficiency of organic foulants by generated ROS would be significantly improved if the reaction space were restricted far below the diffusion length scale of ROS. This phenomenon was reported in previous studies as a spatial confinement effect [19,20]. 

**Figure 8:**
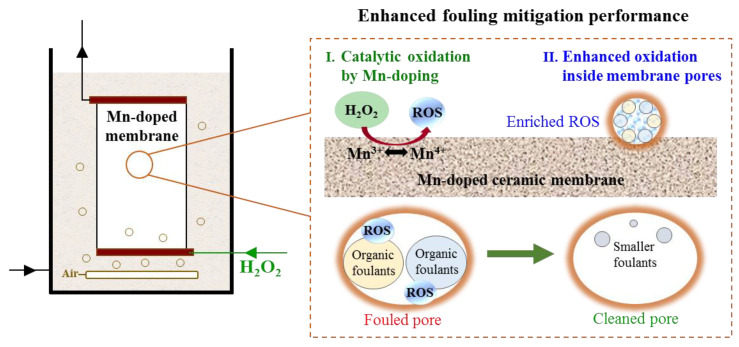
Schematic diagram of the enhanced catalytic removal of organic foulants within Mn-doped membrane via in-situ H_2_O_2_ cleaning.

Organic foulants adsorbed on the membrane/pore surfaces during membrane filtration. When H_2_O_2_ was pumped into membrane pores through the inner cavity, ROS (HO• and ^1^O_2_) were generated on the Mn-doped membrane/pore surfaces and were restricted within the Mn-doped membrane pores (average pore size of 792 nm (Figure S1b)). The oxidation of organic foulants on the Mn-doped membrane/pore surfaces occurred once ROS were generated. As shown in Figure 8, the confined reaction space within the Mn-doped membrane pores shortened the diffusion lengths of ROS and prevented their elimination, leading to enriched ROS within the membrane pores. Because of the substantially reduced diffusion length and concentrated reactants, ROS reacted with organic foulants within membrane pores with much higher efficiency than they did in the bulk phase [19,55]. In this way, organic foulants were efficiently removed, and membrane fouling was well controlled via in-situ H_2_O_2_ cleaning confined in the Mn-doped membrane pores.

Overall, coupling the Mn-doped membrane with in-situ H_2_O_2_ cleaning achieved the best fouling mitigation performance with the lowest TMP increase, the highest TMP recovery efficiency and the fewest DOC in fouling layers, as a result of improved surface properties and confined catalytic oxidation of organic foulants by H_2_O_2_ inside membrane pores.

## 4. Conclusions

In-situ H_2_O_2_ cleaning confined to the inside of the membrane was applied to mitigate membrane fouling in real municipal wastewater treatment. An Mn-doped membrane was used in a membrane bioreactor for wastewater treatment with the aim to improve the membrane catalytic activity toward oxidation of foulants by H_2_O_2_. The Mn-doped membrane with in-situ H_2_O_2_ cleaning demonstrated a superior fouling mitigation performance to that of a pristine ceramic membrane with no harm to the activated sludge. After 24-h filtration, the ΔTMP of the Mn-doped membrane with H_2_O_2_ cleaning was 22.2 kPa, which was 40.5% lower than that of the pristine membrane with H_2_O_2_ cleaning (37.3 kPa). The enhanced fouling mitigation was attributed to Mn doping that ameliorated membrane surface properties and catalyzed oxidation of foulants by H_2_O_2_ confined in the membrane pores. Mn^3+^/Mn^4+^ redox couples in the active layer of the Mn-doped membrane catalyzed H_2_O_2_ decomposition continuously to generate ROS (HO• and ^1^O_2_), which were confined within the membrane pores and efficiently degraded foulants. Overall, coupling the Mn-doped membrane with confined H_2_O_2_ cleaning achieved satisfactory fouling mitigation with a low TMP increase and high TMP recovery efficiency. The findings provide a new strategy for the rational design of antifouling membranes and fouling mitigation processes.

## Data Availability

Not applicable.

## Supplementary Materials

The following are available online at https://www.mdpi.com/article/10.3390/membranes12010021/s1, Figure S1. The pore size distribution of the (a) pristine and (b) Mn-doped membrane layers; Figure S2. H_2_O_2_ decomposition catalyzed by commercial metal oxide particles at a neutral pH. (a) Al_2_O_3_ or SiO_2_; (b) Mn oxides. (Operating conditions: *C*_0_ = [H_2_O_2_]_0_ = 1 mM; *C* is the concentration of H_2_O_2_; *M*_metal oxides_ = 1 g/L except *M*_Mn oxides_ = 0.05 g/L; initial pH = 6.8–7.0.); Figure S3. Water quality of the permeation when membranes were in-situ cleaned with H_2_O or H_2_O_2_. (a) Turbidity, (b) DOC, (c) total nitrogen, (d) total phosphorus; Figure S4. MW distribution of DOC in the feed and permeation during membrane filtration at a flux of 60 LMH without backwash. (a) MW distribution of DOC; (b) integral areas of different MW components; (c) rejection rates of different MW components by membranes; Figure S5. MW distribution of DOC in the physically removable fouling layer after 24 h of filtration; Figure S6. Pure water contact angles of the pristine (a) and Mn-doped membranes (b); Figure S7. XPS spectra of O 1s of the pristine and Mn-doped ceramic membranes; Figure S8. Pure water permeabilities of the pristine membrane and Mn-doped membrane; Table S1. Mixed liquor properties after membrane filtration.
