# Supplementary material for: In-Situ H2O2 Cleaning for Fouling Control of Manganese-Doped Ceramic Membrane through Confined Catalytic Oxidation Inside Membrane
Source: Membranes (Basel). 2021 Dec 24;12(1):21. doi: 10.3390/membranes12010021 (PMC8777854; doi:10.3390/membranes12010021)
Supplement: Supplementary file 1 [file membranes-12-00021-s001.zip › membranes-1525629-supplementary.pdf]

Supplementary Materials

In-Situ H_2O_2 Cleaning for Fouling Control of Manganese-Doped Ceramic Membrane Through Confined Catalytic Oxidation Inside Membrane

Shengyin Tang [1,2], Wanyi Fu [1,*], Tiantian Song [1], Tianhao Tang [1], Li Chen [3], Jianning Guo [4], Slav W. Hermanowicz [2] and Xihui Zhang [1,3*]

[1] Tsinghua-Berkeley Shenzhen Institute, Tsinghua University, Shenzhen 518055, Guangdong, China
[2] Tsinghua Berkeley Shenzhen Institute, University of California, Berkeley, CA 94720, USA
[3] Tsinghua Shenzhen International Graduate School, Tsinghua University, Shenzhen 518055, Guangdong, China
[4] Institute of Information Technology, Shenzhen 518172, Guangdong, China
* Correspondence: XZ, zhangxh@sz.tsinghua.edu.cn; WF, fu.wanyi@sz.tsinghua.edu.cn

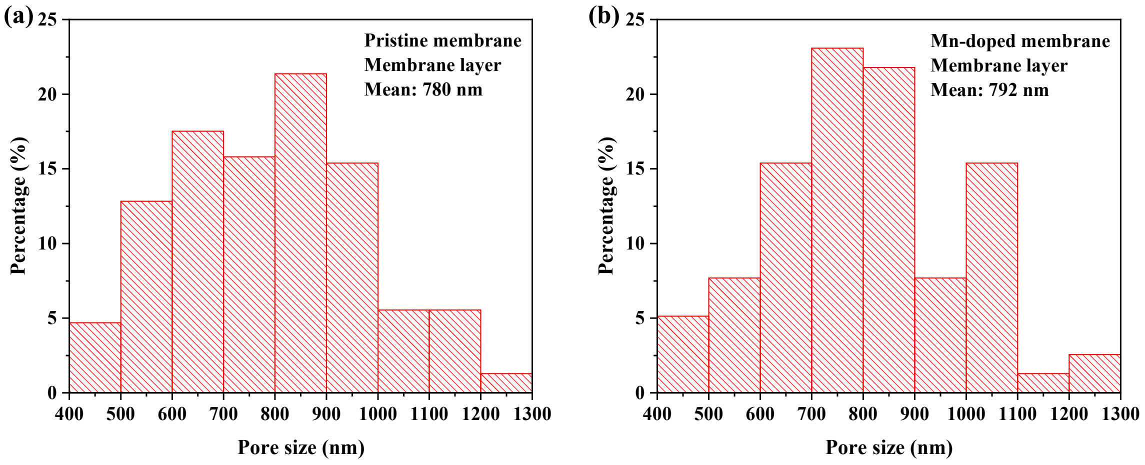

Figure S1. The pore size distribution of the (a) pristine and (b) Mn-doped membrane layers.

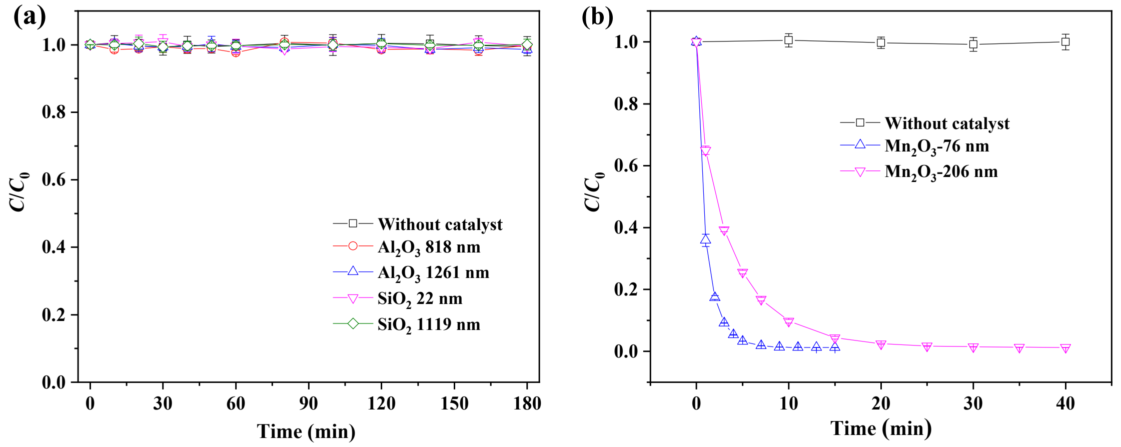

Figure S2. H_2O_2 decomposition catalyzed by commercial metal oxide particles at neutral pH. (a) Al_2O_3 or SiO_2; (b) Mn oxides. (Operating conditions: $C_0 = [H_2O_2]_0 = 1$ mM; C is the concentration of H_2O_2; $M_{\text{metal oxides}} = 1$ g/L except $M_{\text{Mn oxides}} = 0.05$ g/L; initial pH = 6.8-7.0)

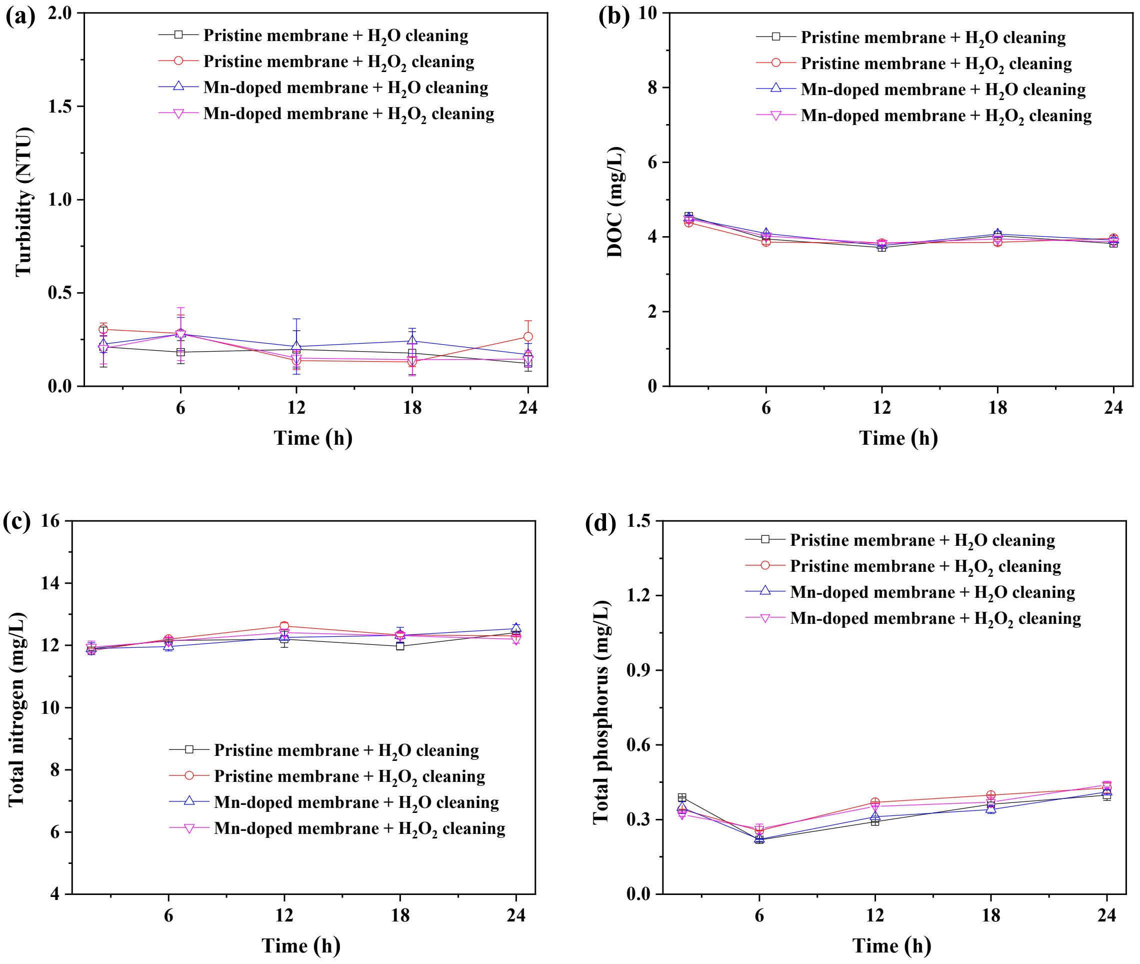

Figure S3. Water quality of the permeate when membranes were *in-situ* cleaned with H_2O or H_2O_2. (a) Turbidity, (b) DOC, (c) total nitrogen, (d) total phosphorus.

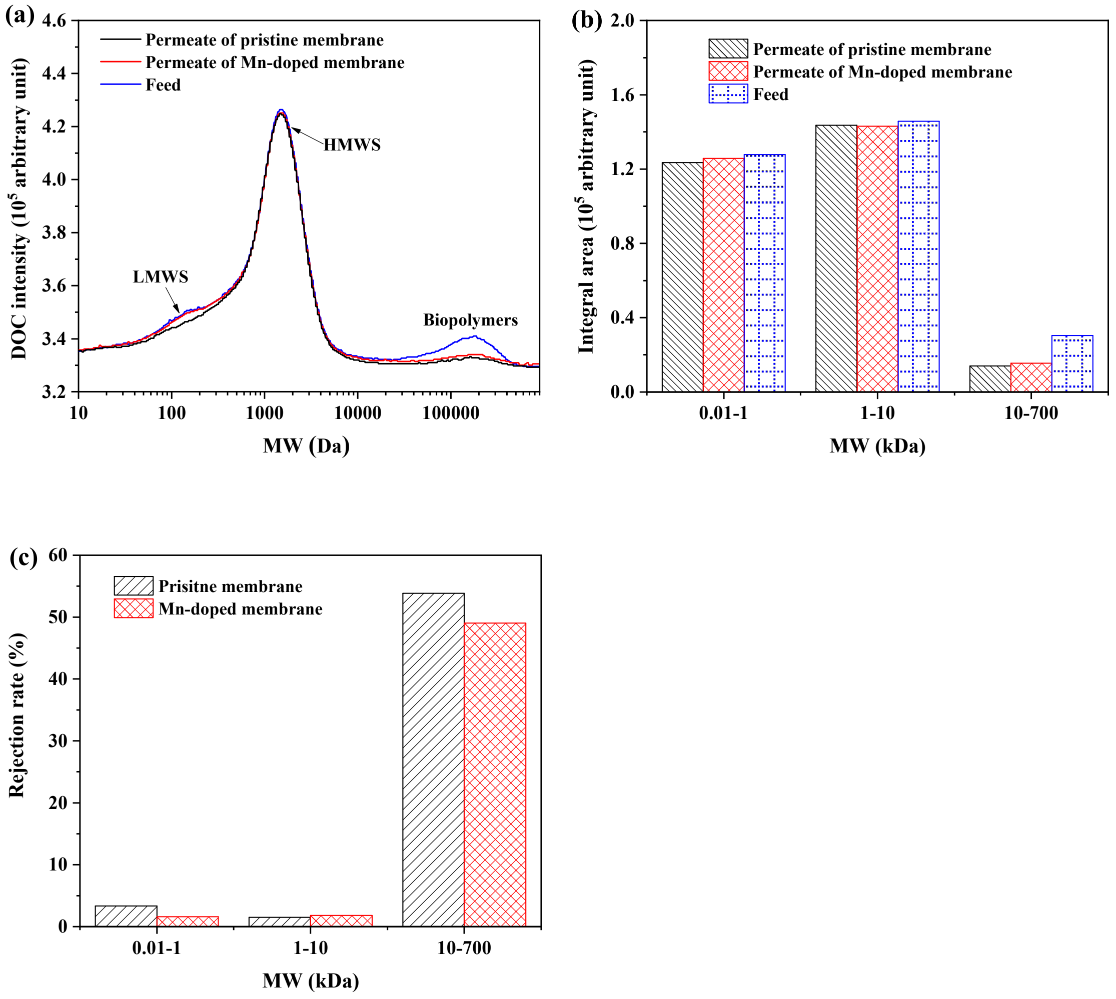

Figure S4. MW distribution of DOC in the feed and permeates during membrane filtration at flux of 60 LMH without backwash. (a) MW distribution of DOC; (b) integral area of different MW components; (c) rejection rates of different MW components by membranes.

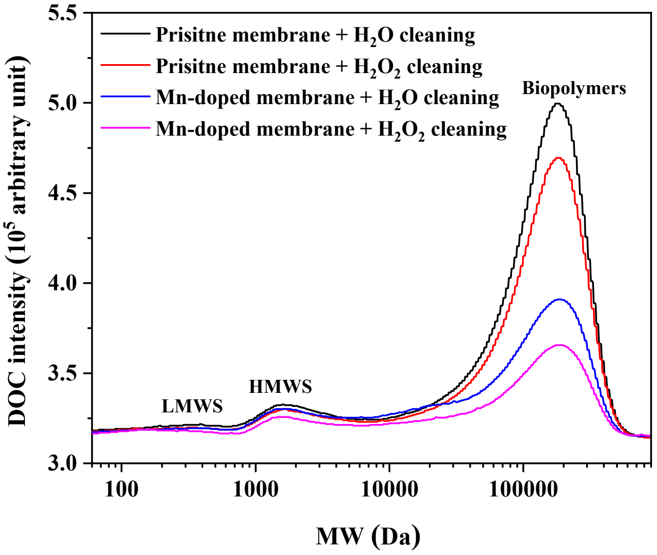

Figure S5. MW distribution of DOC in the physically removable fouling layer after 24 hours filtration.

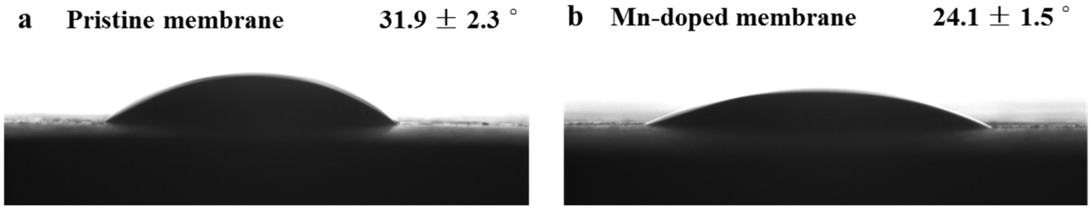

Figure S6. Pure water contact angles of the pristine (a) and Mn-doped membranes (b).

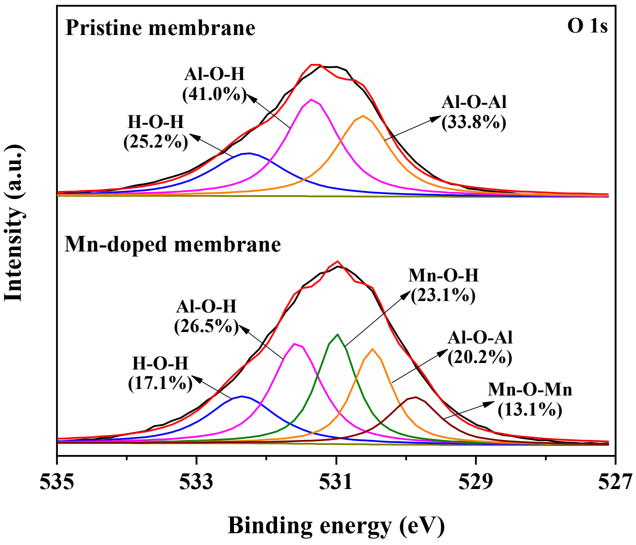

Figure S7. XPS spectra of O 1s of the pristine and Mn-doped ceramic membranes.

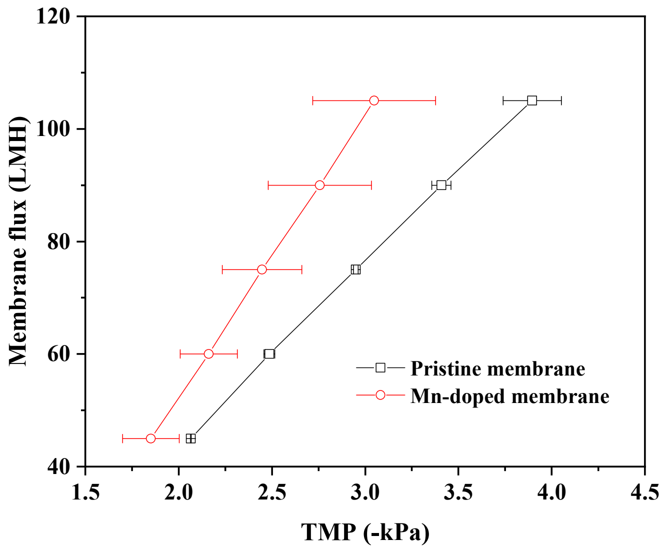

Figure S8. Pure water permeabilities of the pristine membrane and Mn-doped membrane.

Table S1. Mixed liquor properties after membrane filtration.

Mixed liquor properties	Pristine membrane + H_2O cleaning	Pristine membrane + H_2O_2 cleaning	Mn-doped membrane + H_2O cleaning	Mn-doped membrane + H_2O_2 cleaning
DOC (mg/L)	5.5 ± 0.2	5.5 ± 0.1	5.7 ± 0.3	5.6 ± 0.2
MLSS (g/L)	7.2 ± 0.1	7.1 ± 0.2	7.1 ± 0.2	7.0 ± 0.1
SV_{30} (%)	39.0 ± 1.4	38.8 ± 1.8	39.3 ± 1.1	39.1 ± 0.6
Zeta potential (mV)	-12.1 ± 0.8	-12.0 ± 0.7	-12.1 ± 0.3	-11.9 ± 0.3
SOUR (mg O_2/(g MLSS*min))	0.1338 ± 0.0110	0.1326 ± 0.0008	0.1340 ± 0.0122	0.1432 ± 0.0025
pH	6.9 ± 0.1	7.0 ± 0.1	6.9 ± 0.1	7.1 ± 0.1
